# Learning through Osmosis: A global Wikipedia editing course for medical students

**DOI:** 10.15694/mep.2020.000109.1

**Published:** 2020-05-22

**Authors:** Tolga Guven, Carolyn Geraci, Jason Green, Johnathon Neist, Monica Gopalakrishnan, Puja Bhatt, Tanya Cupino, Amin Azzam

**Affiliations:** 1 Charles University; 2 Larner College of Medicine; 3 St. George's University; 4 Medical College of Wisconsin; 5 Saveetha Medical College and Hospital; 6 International American University College of Medicine; 7 Loma Linda University; 8 University of California

**Keywords:** Global health, Curricular innovation, Medical education, Open educational pedagogy, Wikipedia

## Abstract

This article was migrated. The article was marked as recommended.

**Problem:** Wikipedia is a ubiquitous source of information for patients, medical students and junior doctors alike. This is despite medical educators discouraging students from Wikipedia as a source of medical information.

**Intervention:** To address this disconnect, Osmosis’ Director of Open Learning Initiatives created a novel Wikipedia-editing course structured to leverage the global network of Osmosis-subscribing students. The course was entirely video-conference based and lasted 4 weeks in July 2019. Students typically worked on an article by themselves though one article was selected by two students. Towards the end of the course, each student peer-reviewed another student’s edited article.

**Outcomes:**Twelve medical students, from 11 different medical schools across 3 different continents, enrolled in the course and 11 articles were assigned. A total of 8,775 words and 119 higher quality references were added whilst 35 lower-quality references were removed. An exit survey showed students had increased confidence in their ability to contribute to Wikipedia. Students also enjoyed collaborating with a global diversity of peers.

**Lessons Learned:** Numerous students wished that the course had a longer duration. A couple students recommended more groupwork to be incorporated into the course. The global nature of the course meant that time zones proved a challenge to scheduling.

**Conclusion:**Decentralized courses can leverage the large user bases of medical education companies, such as Osmosis, to teach students analytical approaches to online resources as well as improve the quality of publicly available health information on Wikipedia.

## Problem

In the age of digital technology, Wikipedia has emerged as a widely-used resource for health information ( Heilman *et al.*, 2011). After receiving a diagnosis, patients who search online for additional medical information often use Wikipedia for a concise summary of their symptoms or conditions ( Heilman and West, 2015). Like these patients, many physicians and nearly all medical students report using Wikipedia to look up medical information ( Hughes *et al.*, 2009; Allahwala, Nadkarni and Sebaratnam, 2013).

Wikipedia is often presumed to be unreliable because of its open source collaborative editing structure. Many medical educators discourage the use of Wikipedia, and instead direct students to use resources that require paid subscriptions. Since students are consulting Wikipedia despite these admonitions, there is a discontinuity between medical student practices and the prevailing medical education paradigm. As patients frequently cannot access paid medical databases, there is a similar disparity between “physician-facing” and “patient-facing” information sources.

A novel alternative to teaching medical student Wikipedia abstinence is to teach students how to evaluate and improve the quality of articles under an expert facilitator. This confers at least two benefits: 1) students develop critical thinking and reasoning skills when using online resources, and 2) patients, laypeople and future students find more accurate information on Wikipedia. A few early-adopting schools offer courses on medical Wikipedia editing, though the pace of adoption in other institutions has been slow ( Joshi *et al.*, 2019). Limitations include a lack of faculty enthusiasm and insufficient time in the curriculum for an additional course.

In this report, we detail an alternative that bypasses such limitations via a decentralized course. Specifically, we developed an online course, run by a medical school faculty member and a medical librarian at two different institutions, that instructed students on how to 1) evaluate the reliability of a Wikipedia article and the referenced material, 2) assess and incorporate new references, 3) improve poor quality medical articles, and 4) interact through a peer review process. This approach may reduce barriers to training larger numbers of medical students to evaluate and improve health information on Wikipedia, addressing a critical need as the relatively small number of core medical editors continues to decline ( Heilman and West, 2015).

## Intervention

Osmosis is a medical and health sciences education technology platform used by 800,000 active users. In January 2017, Osmosis received grants to promote open educational pedagogy-a curricular strategy where students learn by producing open educational resources ( Hewlett Foundation, 2018). Leveraging the large global network of Osmosis-subscribing medical students, Osmosis’ Director of Open Learning Initiatives created a novel Wiki-editing course structure. The course lasted 4 weeks in the summer of 2019. Students were invited to apply for the course by filling out a GoogleForm, stating their motivation to take part in such a course, as well as prior academic or teaching experience.

The course was run by a faculty member as well as a librarian, and began with an entirely videoconference-based opening session where students were introduced to becoming a Wikipedian in medical school, as well as their peers. Students were then expected to complete nine training modules on the Wiki Education platform (categorized broadly as “the basics of Wikipedia” and “editing Wikipedia as a health-professional”) and identify a single Wikipedia page to edit for the duration of the course by the second session. Students could work on a page individually, or in groups with clearly defined roles. During the second session, work plan strategies were discussed as well as expectations for weekly work-in-progress (WIP) videoconference meetings and a peer-review in the penultimate week. From this discussion a list of expectations for WIP meetings and the peer-review were drawn up and posted on the WikiEdu platform for guidance and accountability. Additionally, JN, the librarian, had a presentation on “Library resources, searching strategies and techniques” to inform students on how to assess as well as find reliable sources to edit their Wikipedia articles. The librarian remained available throughout the course for consultation on information seeking strategies, as well as credibility of online resources. Students were expected to develop a workplan and to post it on the “Talk” page of their chosen Wikipedia article, where the Wikipedia community could interact with and provide feedback on their proposed changes throughout the month. Students working in groups had to detail which individual was responsible for which section for a transparent and accountable collaboration. There were 4 weekly work-in-progress meetings as well as a peer-review in the penultimate week, which was then followed up by a response to the peer-review in the final week, allowing students to learn about the peer-review process.

To have the highest potential impact, students were advised to select pages that were rated top or high importance by WikiProject Medicine, or other health related WikiProjects, as well as relatively low on the Wikipedia’s quality rating (C or Start quality). All details of the course structure are available on Wikipedia itself ( Azzam, 2019). To assess our course, we invited students to complete a 19-item post course survey about their experiences. The University of California, San Francisco internal review board approved this study of our novel curricular structure.

## Outcomes

A total of 29 students submitted the first part of a 2-part application. Of these, 19 completed part 2 and 18 were accepted. One individual who completed the application was not accepted and one individual who did not formally complete the application process was enrolled in the course. A total of 12 students attended orientation, of which 11 completed all training modules and posted workplans on Wikipedia. Though only 10 articles were assigned, students enrolled in the course voluntarily edited an additional 18 articles. Extracurricular editing typically involved small improvements in grammar and punctuation. To the assigned articles, a total of 8,775 words and 119 higher-quality references were added whilst 35 lower-quality references were removed. The 11 assigned Wikipedia pages were viewed 175,022 times during the course. A total of 10 students submitted peer reviews of an article edited by a fellow student and completed the course in its entirety. Three of the 11 students continued to edit Wikipedia after the course was completed. Table 1 provides a detailed breakdown of student impact on Wikipedia’s content.

**Table 1.** Summary of articles edited during the course

Importance Score	WikiProject ^a^	Associated Articles	References Added	Student Editors	Total Editors ^b^	Page Views per Day ^c^
**Top**	Pharmacology	1	18	1	2	389
**High**	Medicine	7	60	9	39	5,041
	Neuroscience	2	37	3	9	785
	Pharmacology	2	37	3	7	633
	Viruses	1	37	2	6	508
**Mid**	Anatomy	1	16	1	4	168
	Chemicals	1	37	2	6	508
	Medicine	4 ^*^	37	3	15	1,956
	Microbiology	1	9	1	8	1,177
	Physiology	2 ^*^	-35	2	9	2,984
	Sexology and Sexuality	1	9	1	7	964
**Low**	Medicine	6 ^**^	0	1	13	2,507
	Neuroscience	1 ^**^	0	1	2	675
	Women’s Health	1	9	1	7	964

^a^ Approximately 37% of articles edited by students during the course were assigned to more than one WikiProject. Unless otherwise noted, numbers reflect on the changes made by students enrolled in the Osmosis Wiki Education course.

^b^ This is the sum of all unique editors, including students.

^c^ Daily world-wide page views for the edited articles between July 7 and August 3, 2019.

* One or more articles were edited outside of course assignments.

** All articles were edited outside of course assignments.

**Figure 1.**
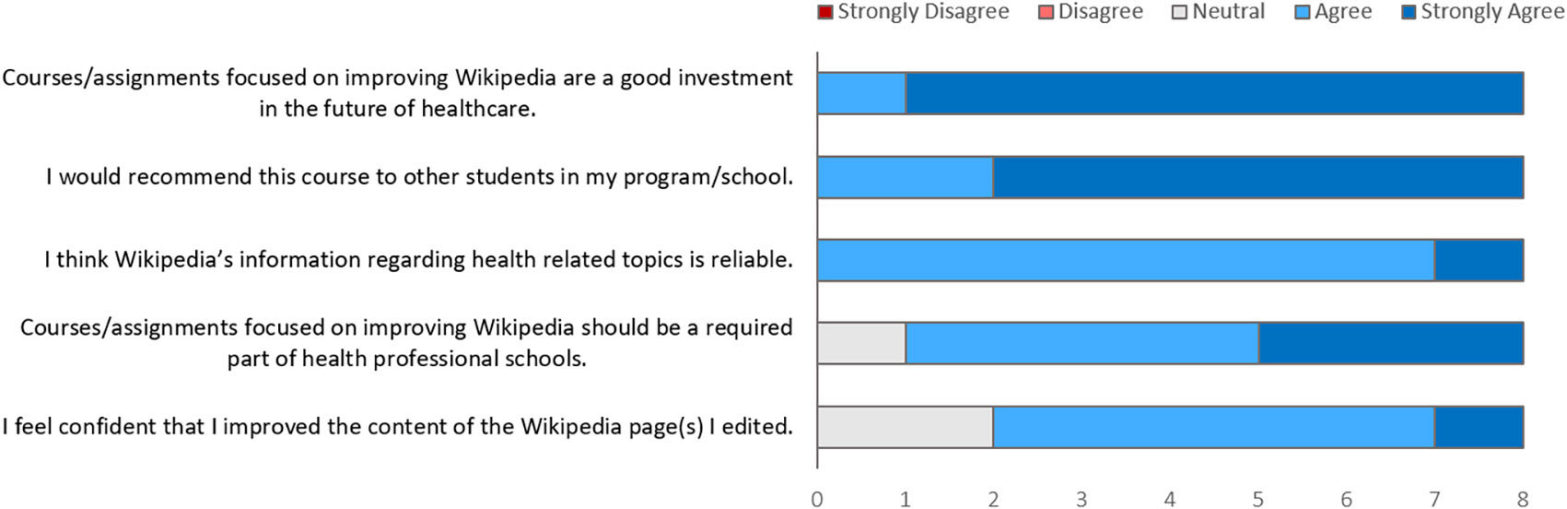
Summary of student’s views on Wikipedia and the course from post-course Survey

We queried all 10 students who completed the course and 9 completed our survey (90% response rate). Student responses were overwhelmingly positive--all agreed that courses such as these are a good investment into the future of healthcare. One student’s responses to the quantative section of the survey were discounted due to suspected human error, as they were in clear contradiction to what the student filled out in the short-answer form. Figure 1 summarizes key quantitative survey results.

Students felt a sense of ownership towards the work they were producing on Wikipedia and reported a growing trust in health-related content on Wikipedia. To evaluate free text survey items, we consolidated students’ responses into themes. Table 2 summarizes these themes with representative exemplar quotes.

**Table 2.** Summary of students’ free text responses to the end course survey

Question	Response Theme	Representative Quotes
For what reason(s) did you decide to take this course?	Self-improvement	• I have been a copy-editor for 3 years, but I wanted to improve my skills and start improving/creating more content. • I like the chance to learn new skills and learn actively.
	Novelty	• I just wanted to try something out of my monotonous routine of college life, so I came across this course and immediately took it. • I thought it would be a new challenge.
	Access to information	• I wanted to help underserved populations have access to accurate healthcare information. • I love Wikipedia; ever since high school, it has been the default reference for all things in my life ranging from horror movie summaries to medical school questions. It is concise (usually), well formatted, and incredibly easy to navigate. Also, a joy to interact with. How could I pass up the opportunity to get involved with a community and resource that had been there for me through so much of my life?
Have your opinions on Wikipedia changed since completing this course? If so, how?	Increased trust in Wikipedia	• I did not have any opinion against Wikipedia, but I have heard doctors and few students in my college tell it’s not a reliable source of information as it can be edited by anyone. But now I think I can tell them that is not the case. • Yes, I learned a lot about the grading scale and which articles to “trust” vs those to be more wary about. • I certainly have more confidence in the community! I’m not sure how much may be skewed by my limited exposure to the Wikiproject Medicine community, but where before I had a general inclination to trust the skeletons of Wikipedia articles based on trust in communities if not individuals, I now very much feel more comfortable believing in the mission and product that Wikipedia puts forward.
	Contribution	• Yes! I’m willing to support Wikipedia financially. I feel a sense of ownership to improve Wikipedia articles. • Not of Wikipedia itself as I always valued open access to information, but my ability to contribute to it in a meaningful way.
What do you feel is the most valuable thing you learned from this course?	Collaboration	• How to work as a team on articles. • I learnt how to communicate to new people from various places, how to edit pages, the technical side of it. And lastly how to comment on peer talk pages. • Learning that this was global effort. Before our first zoom meeting I read up on some of the course work and thought it’s great that there is some effort to improve Wikipedia but had no idea that it was done on such a global scale, that is truly inspiring.
	Initiative	• I feel empowered to “be bold” and make the internet a better place for important health information. • Assert yourself in good faith! Not necessarily something I learned as a novel concept, but one I find myself continuing to discover applications for in a growing number of areas.
This is the first time we have offered this course to a distributed network of medical students. Please comment on how this course structure impacted your experiences (either positively or negatively).	Enjoying the diversity	• I really liked having medical students from across the world in the course. • It was really great to have a chance to talk and learn from people with many different backgrounds. Hearing perspectives from all the students was really an eye-opening experience. There was a sense of community knowing that we all were working towards the same goal even if on different topics. • I sincerely enjoyed working with peers from different institutions and stages in medical school. It was encouraging to see the diversity of people involved. If it had not been offered in this way, I doubt if I would ever have the opportunity to participate in a course like this at my institution.
Did this course influence your professional identity? If so describe how.	Physician as educator	• I’m not sure it influenced my professional identity, but it did make me much more aware of the power of medical students to enact positive change in a real way. Also, I’m highly interested in incorporating Wikipedia editing into any course that I teach in the future.
	Spokesperson	• I’m more comfortable talking about Medical Wikipedia as a legitimate resource and about my role in helping to improve it. • Yes, I now feel empowered to educate my mentors and attendings on how people are using the internet for health information.
	Communication skills	• I want to maintain my wiki editing skills and develop my abilities to communicate key ideas. I also want to encourage peers and students to feel safe enough to offer suggestions and make edits, in accordance with style guides and well cited. • I’m now more considerate and aware of how medical information sounds/can be conveyed to patients/the general public.

## Lessons Learned

The post-course survey provided valuable feedback on how students felt the course could be improved. Numerous student responses recommended that extending the course would improve student experience. One student recommended more time specifically for researching which article to select. A potential benefit for allowing a longer selection period could be increased motivation towards their chosen topic.

A couple students suggested more collaborative editing efforts (e.g. multiple students working together on a single article). It’s worth noting that students were allowed to work in groups during this course, but most opted not to do so. This may be due to a limited time getting to know each other before starting the editing process. Future iterations of this course could consider how to implement more groupwork into the course structure.

Students were encouraged on to work on lower-rated articles. Low quality articles typically have few contributing editors, which diminishes opportunities for discussion during coursework. Another drawback was less engagement from the Wikipedia community.

Enrolled students hailed from 3 different continents, leading to challenges associated with time-zone coordination (e.g. what time to hold video-conference sessions). We also limited eligibility to Osmosis-subscribing medical students, which may not accurately represent the global pool of medical students.

## Conclusion

While learning how to improve Wikipedia, students developed critical thinking through evaluating referenced material and participating in peer review. These future health practitioners also learned how to navigate Wikipedia’s quality ratings. This skill can be shared with future patients to enable independent evaluation of health information on Wikipedia. The outcome of greatest potential public health impact is the direct improvements to health-related Wikipedia content, which may be read by patients, laypeople and future students. By opening a course to a global cohort, students from underserved regions and countries will be able to network and learn with peers from wealthier areas. Together, these outcomes serve to bridge a gap in the flow of information between the medical profession and the communities in which we serve, in both the privileged and underdeveloped world.

## Take Home Messages

• Wikipedia is widely-used as a source for health information by medical students, junior doctors and patients.
• Global online learning platforms can be leveraged to improve the quality of health-related information on Wikipedia.
• Through their editing medical students learn and develop the ability to evaluate the quality of online information.
• Wikipedia-editing pushes students to express their medical knowledge in accessible and simple terms.
• Students around the world are able to collaborate and learn from each other’s unique experiences.

## Notes On Contributors

**Tolga Kamil Guven** is a final year medical student at Charles University in Prague. His interests include surgery, medical education and open-access to medical information. ORCID iD: https://orcid.org/0000-0002-7733-4266

**Carolyn Geraci** is a medical student at the University of Vermont - Larner College of Medicine.

**Jason Green** is a medical student at St. George’s University.

**Johnathon Neist** is a librarian at the Medical College of Wisconsin.

**Monica Gopalakrishnan** is an intern at Saveetha Medical College and Hospital, India.

**Puja Bhatt** is a medical student at the International American University College of Medicine.

**Tanya Cupino**, PhD is a second year medical student at Loma Linda University in California, USA. ORCID iD: https://orcid.org/0000-0002-4424-7473

**Amin Azzam** is a full professor at 3 San Francisco bay area health professional schools: 1) University of California, San Francisco School of Medicine; 2) University of California, Berkeley School of Public Health; and 3) Samuel Merritt University. He is also a pedagogical consultant on two grants from the Hewlett Foundation to Knowledge Diffusion, Inc (DBA Osmosis). The grants are to promote open educational pedagogy. His role is to help other health professional schools (that are part of the network using Osmosis tools) design and launch Wikipedia-editing assignments and/or courses at their schools. ORCID iD: https://orcid.org/0000-0002-7024-7450

## Declarations

At the time of this work, Dr. Azzam was a pedagogical consultant on a Hewlett Foundation sponsored grant to Osmosis to promote Open Educational Practice (OEP) through scalable technology. This involved working with faculty at other medical and health professional schools to create Wikipedia-editing assignments and/or courses.
