# Supplementary material for: Learning through Osmosis: A global Wikipedia editing course for medical students
Source: MedEdPublish (2016). 2020 May 22;9:109. [Version 1] doi: 10.15694/mep.2020.000109.1 (PMC10702862; doi:10.15694/mep.2020.000109.1)
Supplement: Peer review history

# Reviewer report: Reviewer response for version 1

**DOI:** 10.21956/mep.20024.r30568

**Published:** 2020-06-11

**Authors:** Hebat Allah A. Amin

**Affiliations:** 1 Department of Pathology, Helwan University, Faculty of Medicine

This review has been migrated. The reviewer awarded 4 stars out of 5 An innovative idea with multiple outcomes. It enhances international, team work between the students and drives critical thinking.Despite, refining and upgrading a mega source as Wikipedia is not expected from rhis limited case study, however, I hope this experience can grow.

Reviewer Expertise:

NA

No decision status is available

# Reviewer report: Reviewer response for version 1

**DOI:** 10.21956/mep.20024.r30572

**Published:** 2020-06-02

**Authors:** Aathithyaa Selvaamani

**Affiliations:** 1 Saveetha medical college hospital

This review has been migrated. The reviewer awarded 5 stars out of 5 Excellent work..

Reviewer Expertise:

NA

No decision status is available

# Reviewer report: Reviewer response for version 1

**DOI:** 10.21956/mep.20024.r30571

**Published:** 2020-06-02

**Authors:** P Ravi Shankar

**Affiliations:** 1 American International Medical University

This review has been migrated. The reviewer awarded 4 stars out of 5 This is an interesting article describing an international collaboration to edit Wikipedia medical pages. Students from a number of countries were able to collaborate using internet technologies. The process, outcomes, discussion, conclusion and take-home messages are well-written. The paper is well structured. The small number of students involved could be a limitation. I remember a visit by James Heilman to Nepal nearly ten years ago to explore the possibility of translating some Wikipedia pages to Nepali. He is an excellent source of information about Wikipedia and the philosophy behind the foundation. Wikipedia is a widely used information source and contrary to expectations often provides good quality information. The number of editors and others working on a topic directly contributes to the information quality. The possibility of editing out incorrect information is a powerful feature of Wikipedia. Open editing is a new concept and still misunderstood by many. The fact that Wikipedia is free to access ensures its wide use by patients and students. I would be interested in knowing which specific pages were edited during this project. It will also be interesting to know how the authors intend to carry this project forward.

Reviewer Expertise:

NA

No decision status is available

# Reviewer report: Reviewer response for version 1

**DOI:** 10.21956/mep.20024.r30567

**Published:** 2020-05-26

**Authors:** Maria de los Angeles Fernandez-Altuna

**Affiliations:** 1 Universidad Nacional Autonoma de Mexico Facultad de Medicina

This review has been migrated. The reviewer awarded 2 stars out of 5 The idea reported in this paper is very interesting but this kind of sources should not be used as primary sources for medical students. They should know about them but is better to get informed in more academical platforms. The number of participants were reduced

Reviewer Expertise:

NA

No decision status is available

# Reviewer report: Reviewer response for version 1

**DOI:** 10.21956/mep.20024.r30566

**Published:** 2020-05-24

**Authors:** Nandalal Gunaratne

**Affiliations:** 1 Faculty of Medicine, Wayamba University of Sri Lanka

This review has been migrated. The reviewer awarded 2 stars out of 5 This seems to be an idea that has to be cultivated to bear a fruit. To make an international collaborative effort is a task that will need a lot of doing. This number of students is unconvincing to say the least.But I hope and pray it will indeed become an active snowballing effort by the young minds to create an open access knowledge base that is vibrant and upto date.

Reviewer Expertise:

NA

No decision status is available

# Reviewer report: Reviewer response for version 1

**DOI:** 10.21956/mep.20024.r30570

**Published:** 2020-05-23

**Authors:** Richard Hays

**Affiliations:** 1 James Cook University

This review has been migrated. The reviewer awarded 2 stars out of 5 This paper reports an interesting idea, but the scale required to make a huge, open-source repository a reliable source of information is huge - likely to be well beyond involving even lots of students. Maintaining currency would also be a huge task. The same energy may be better devoted to both searching the primary literature and contributing to Cochrane databases. I can only reaffirm for students that searching only such databases for current information In health care is very unwise. As a learning task in critical appraisal, there is more value. I also like the idea of open-sourcing information so that patients find better information. I also have concerns that the paper reports very small numbers of participants, no real evaluation and has the support of a commercial software developer (this is acknowledged). A much larger, better controlled and evaluated study is needed before much can be said. It may have been better to talk more generically of editing software. It therefore reads a little like an advertisement, so in its present form I do not think that this is a great contribution to an academic journal.

Reviewer Expertise:

NA

No decision status is available

# Reviewer report: Reviewer response for version 1

**DOI:** 10.21956/mep.20024.r30573

**Published:** 2020-05-22

**Authors:** John Sandars

**Affiliations:** 1 Edge Hill University

This review has been migrated. The reviewer awarded 5 stars out of 5 This is a really interesting innovative collaborative multi-national approach to teaching and learning. I like the combination of enhanced learning as a process of doing a task (the editing and its associated activities) and learning as producing an outcome (the updated Wikipedia content ). This could be a demonstrator pilot for a much larger future international collaborative approach to enhance teaching and learning.

Reviewer Expertise:

NA

No decision status is available

# Reviewer report: Reviewer response for version 1

**DOI:** 10.21956/mep.20024.r30569

**Published:** 2020-05-22

**Authors:** Virginia Randall

**Affiliations:** 1 Uniformed Services University of the Health Sciences

This review has been migrated. The reviewer awarded 5 stars out of 5 What a wonderful idea - students collaborating across cultures and learning by doing AND improving the information in Wikipedia. This is a model for design of other course work.

Reviewer Expertise:

NA

No decision status is available
